# The value of tumor deposits in evaluating colorectal cancer survival and metastasis: a population-based retrospective cohort study

**DOI:** 10.1186/s12957-022-02501-9

**Published:** 2022-02-21

**Authors:** Wenhao Wu, Shun Zeng, Xianbin Zhang, Peng Liu, Tong Qiu, Shulin Li, Peng Gong

**Affiliations:** 1 Department of General Surgery & Carson International Cancer Research Center, Shenzhen University General Hospital and Shenzhen University Clinical Medical Academy, Xueyuan Road 1098, Shenzhen, 518055 China; 2 Guangdong Key Laboratory of Regional Immunity and Diseases, Shenzhen University Health Science Center, Xueyuan Road 1066, Shenzhen, 518060 China; 3 Guangdong Key Laboratory for Biomedical Measurements and Ultrasound Imaging, School of Biomedical Engineering, Shenzhen University Health Science Center, Xueyuan Road 1066, Shenzhen, 518060 China; 4 Shenzhen University Clinical Medical Academy, Shenzhen, China

**Keywords:** Colorectal cancer (CRC), Tumor deposits (TDs), TNM staging, Prognosis, Distant metastasis, Survival outcomes

## Abstract

**Background:**

The role of tumor deposits (TDs) in TNM staging of colorectal cancer is controversial, especially the relationship with distant metastasis.

**Purpose:**

This study aimed to determine the effect of TDs on the survival of colorectal cancer and the occurrence of distant metastasis and to determine whether TDs (+) patients behaved similarly to stage IV patients.

**Methods:**

A retrospective analysis of CRC patients from two large independent cohorts from the Surveillance Epidemiology and End Results (SEER) database (*n* = 58775) and the First Affiliated Hospital of Dalian Medical University (*n* = 742).

**Results:**

Univariate logistic analyses revealed that TDs are an independent predictor of liver metastasis [*p* < 0.001; odds ratio (OR): 5.738; 95% confidence interval (CI): 3.560–9.248] in the First Affiliated Hospital of Dalian Medical University’s patients. Meanwhile, TDs are also an independent predictor of isolated organ metastasis [*p* <0.001; odds ratio (OR): 3.028; 95% confidence interval (CI): 2.414–3.79; multiple organ metastases [*p* < 0.001; odds ratio (OR): 4.778; 95% confidence interval (CI): 4.109–5.556]; isolated liver metastasis [*p* < 0.001; odds ratio (OR): 4.395; 95% confidence interval (CI): 4.099–4.713] and isolated lung metastasis [*p* < 0.001; odds ratio (OR): 5.738; 95% confidence interval (CI): 3.560–9.248] in the SEER database. Multivariate analyses suggested TDs are an independent poor prognostic factor for distant metastasis (*p* <0.001).

**Conclusions:**

Our results have shown that compared with patients with negative TDs, CRC patients with positive TDs are more likely to develop distant metastasis. Patients categorized as T4aN2bM0 TDs (+) and T4bN2M0 TDs (+) have a similar prognosis as those with stage IV, and hence these patients should be classified as stage IV.

**Supplementary Information:**

The online version contains supplementary material available at 10.1186/s12957-022-02501-9.

## Introduction

It is estimated that by 2021, there will be 150,000 new cases of colorectal cancer (CRC) and 54,000 deaths in the USA, ranking third in both morbidity and mortality [1]. In China, the latest cancer statistics reported by the National Cancer Center in 2019 show that the incidence of colorectal cancer in our country is ranked third, the mortality rate is ranked fifth, and the mortality rate is generally on the rise [2]. In recent years, great progress has been made in the early diagnosis and treatment of colorectal cancer, but the 5-year survival rate of colorectal cancer remains poor, mainly because most of the patients are diagnosed at a terminal stage or with distant metastasis [3]. The prognosis of colorectal cancer depends on different clinical and pathological factors, many of which have been incorporated into different staging systems [4]. Tumor staging is the basis of cancer therapy, and the TNM staging system, based on histopathological and radiological classification methods, is currently considered the gold standard for various tumors staging [5]. After diagnosing colorectal cancer, detailed staging can enable patients to benefit from more precise treatment methods (the detailed TNM staging system information is shown in the Additional file 1: Table S1). If we can recognize early that the patient has metastasized, we can start adjuvant treatment to improve the success rate of surgery.

Tumor deposits are defined as isolated tumor foci found in the pericolonic or perirectal fat or the adjacent mesentery (mesocolonic fat) away from the invasive margin of the tumor without evidence of residual lymphatic tissue [6]. TDs are significantly correlated with poor prognosis after colorectal cancer surgery [7, 8]. Whether to consider TDs as positive lymph nodes in determining the TNM staging of colorectal cancer has been widely debated for many years, which has led to modifications and changes in subsequent versions of the TNM staging system. Both 7th and 8th AJCC (American Joint Committee on Cancer) TNM staging classified regional LNM-negative, TDs-positive pT lesions as N1c [9]. Recent studies have shown that in the TNM staging system, TDs should be counted as metastatic lymph nodes [10–13]. The existence and quantity of TDs are strongly correlated with the prognosis of colorectal cancer patients [14–17], and more and more people support that the TDs as a sign of distant metastasis.

Part of colorectal cancer patients enrolled in the Surveillance, Epidemiology, and End Results (SEER) database and The First Affiliated Hospital of Dalian Medical University were included in this study to explore the impact of TDs on the survival and prognosis of patients and the relationship between TDs and distant metastasis.

## Patients and methods

### Data collection and definition

The treatment data of stage I–IV CRC patients in the two groups were analyzed retrospectively. The first set of data comes from the SEER database. Between 2010 and 2012, 80,428 patients with CRC underwent therapeutic resection. The second set of data comes from the Department of General Surgery, The First Affiliated Hospital of Dalian Medical University, and selected 853 CRC patients who underwent surgical treatment from January 2011 to December 2015. The diagnosis of all CRC patients and the definition of TDs were determined according to the AJCC 7th TNM.

**Fig. 1:**
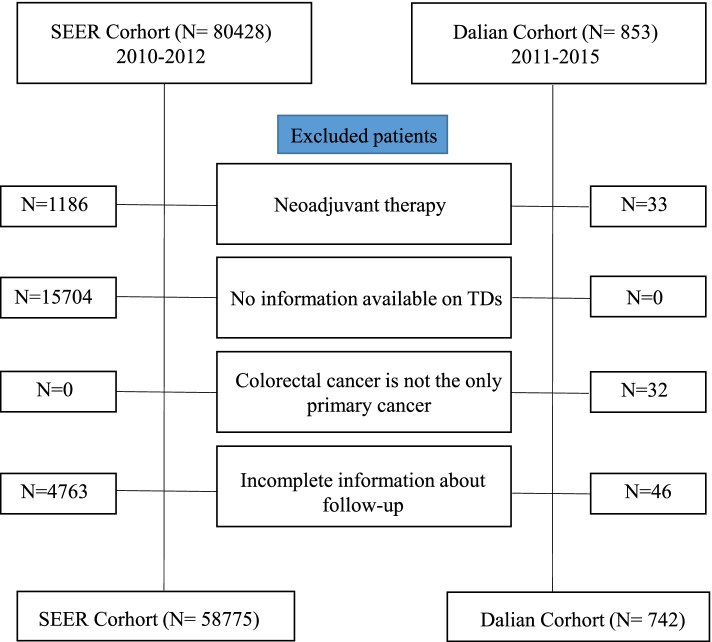
Flowchart of patient selection

To establish a prognostic model for predicting survival and metastasis, we reviewed the clinicopathological features of all patients, including age, sex, tumor location, tumor grade, perineural invasion, presence of TDs, etc. Exclusion criteria included (1) neoadjuvant therapy; (2) no information available on TDs; (3) colorectal cancer is not the only primary cancer; (4) patients with incomplete information about follow-up (Fig. 1).

### Study outcomes

The end point of the study was patient death and distant metastasis was observed. Patients’ overall survival (OS) was defined as from CRC diagnosis to death. Distant metastasis was defined as the presence of liver, lung, bone, or brain metastases during or after CRC diagnosis.

### Statistical analysis

All statistical analyses were performed using SPSS 26.0. The chi-square test was used to analyze categorical variables' demographic and clinical characteristics. The logistic regression coefficients were used to estimate the odds ratios (OR) for the relationship between TDs and distant metastasis patterns. The Kaplan-Meier curve was used to calculate the survival rate, and the log-rank test was used to assess the difference. Calculated hazard ratio (HR), 95.0% confidence interval (CI), and Cox proportional hazards model were used for univariate and multivariate analysis. In multivariate analyses, the clinicopathological characteristics with *p* < 0.05 in univariate analysis were included to determine independent prognostic factors. Significance was set at *p* < 0.05.

## Results

### Characteristics of patients

We extracted two sets of data, including 58,775 and 742 CRC patients, respectively. Overall, the TDs-positive patients in the SEER database and the First Affiliated Hospital of Dalian Medical University were 12.07% (*n* = 7096) and 27.90% (*n* = 207), respectively.

In the SEER database, the most common sites of metastasis are the liver (8.29%, *n* = 4874), followed by the lung (1.74%, *n* = 1024), bone (0.27%, *n* = 159), and brain (0.09%, *n* = 54). Only liver metastasis (11.46%, *n* = 85) was shown in the data from The First Affiliated Hospital of Dalian Medical University. Table 1 shows detailed clinicopathological data from the SEER Database and The First Affiliated Hospital of Dalian Medical University.

**Table 1:** Baseline demographic and related clinical characteristics for patients diagnosed with colorectal cancer

Characteristics	No. of colorectal cancer patients SEER (2010–2012)			No. of colorectal cancer patients Dalian (2011–2015)		
	With TDs (*N* = 7096, 12.07%)	Without TDs (*N* = 51679, 87.92%)	*p* value	With TDs (*N* = 207, 27.90%)	Without TDs (*N* = 535,72.10%)	*p* value
Age, in years			< 0.001			0.826
< 65	3263 (13.80)	20379 (86.20)		115 (27.58)	302 (72.42)	
≥ 65	3833 (10.91)	31300 (89.09)		92 (28.31)	233 (71.69)	
Sex			0.217			0.011
Male	3450 (11.58)	25530 (88.42)		137 (31.42)	299 (68.58)	
Female	3046 (10.51)	26149 (89.49)		70 (22.89)	236 (77.11)	
Race			0.230			
White	5687 (12.05)	41510 (87.95)		—	—	
Black	810 (12.29)	5780 (87.71)		—	—	
Other	559 (12.00)	4389 (88.00)		—	—	
AJCC			< 0.001			< 0.001
I	66 (0.47)	13816 (99.53)		3 (3.33)	87 (96.67)	
II	582 (3.02)	18705 (96.98)		58 (16.76)	288 (83.24)	
III	3855 (20.63)	14830 (79.37)		109 (44.49)	136 (55.51)	
IV	2573 (37.28)	4328 (62.72)		37 (60.66)	24 (39.34)	
T stage			< 0.001			< 0.001
T1	104 (1.40)	7318 (99.60)		0 (0)	19 (100)	
T2	258 (2.73)	9198 (97.27)		8 (9.30)	78 (91.70)	
T3	4087 (12.55)	28487 (87.45)		6 (10.71)	50 (89.29)	
T4	2647 (28.39)	6676 (71.61)		193 (30.22)	388 (69.78)	
N stage			< 0.001			<0.001
N0	823 (2.39)	33611 (97.61)		70 (15.35)	386 (84.65)	
N1	3095 (20.92)	11695 (79.08)		94 (42.53)	127 (57.47)	
N2	3178 (33.27)	6373 (66.73)		43 (66.15)	22 (33.85)	
M stage			< 0.001			<0.001
M0	4523 (8.72)	47351 (91.28)		151 (22.27)	527 (77.73)	
M1	2573 (37.28)	4328 (62.72)		56 (87.50)	8 (13.50)	
Primary site			< 0.001			0.199
Colon	5509 (11.84)	40929 (88.16)		99 (25.85)	284 (74.15)	
Rectum	1587 (12.97)	10750 (87.03)		108 (30.08)	251 (69.92)	
Grade			< 0.001			
I	274 (5.99)	4300 (94.01)		—	—	
II	4396 (10.33)	38149 (89.67)		—	—	
III	1909 (19.90)	7683 (80.10)		—	—	
IV	517 (25.05)	1547 (84.95)		—	—	
Perineural invasion			< 0.001			
None	4761 (9.11)	47473 (90.89)		—	—	
Yes	2335 (35.70)	4206 (64.30)		—	—	
Liver metastasis			< 0.001			< 0.001
None	5409 (10.04)	48492 (89.96)		153 (23.29)	504 (76.71)	
Yes	1687 (34.61)	3187 (65.39)		54 (63.53)	31 (36.47)	
Lung metastasis			< 0.001			
None	6736 (11.66)	51015 (88.34)		—	—	
Yes	360 (35.16)	664 (64.84)		—	—	
Bone metastasis			< 0.001			
None	7032 (12.00)	51584 (88.00)		—	—	
Yes	64 (40.25)	95 (59.75)		—	—	
Brain metastasis			0.002			
None	7082 (12.06)	51639 (87.94)		—	—	
Yes	14 (25.93)	40 (74.07)		—	—	

### TDs associated with OS in SEER cohort

**Fig. 2:**
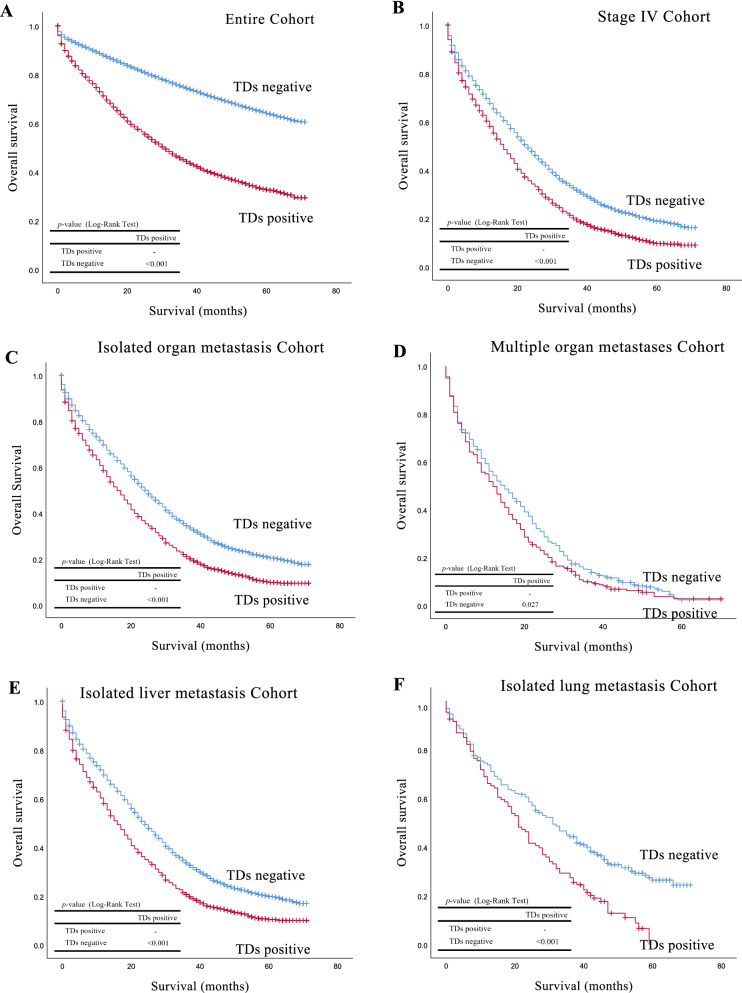
The Kaplan-Meier and log-rank test of overall survival (OS) based on the different cohort. The patients with TDs showed significantly shorter OS than patients without TDs. **A** Entire cohort. **B** Stage IV cohort. **C** Isolated organ metastasis cohort. **D** Multiple organ metastases cohort. **E** Isolated liver metastasis cohort. F Isolated lung metastasis cohort

Compared to the patients with negative TDs, the patients with positive TDs were significantly associated with worse OS in the entire cohort (54.37 vs 36.56 months, *p* < 0.001) and stage IV cohort (29.36 vs 22.21 months, *p* < 0.001). (Survival information for stage I–III cohort is shown in the Additional file 2: Fig. S1). In order to better investigate the significance of TDs in stage IV patients, we divided the stage IV patients into isolated organ metastasis cohort and multiple organ metastases cohort, and the isolated organ metastasis group was further divided into isolated liver metastasis group and isolated lung metastasis group (survival information for isolated bone, brain metastasis cohort is shown in the Additional file 3: Fig. S2). The results showed that TDs-positive patients still showed worse OS in the isolated organ metastasis cohort (30.59 vs 22.55 months, *p* < 0.001) and multiple organ metastases cohort (18.92 vs 16.18 months, *p* = 0.027). Similarly, the same results were obtained in the isolated liver metastasis cohort (30.59 vs 22.55 months, *p* < 0.001) and isolated lung metastasis cohort (30.59 vs 22.55 months, *p* < 0.001, Fig. 2).

### TDs was an independent prognostic factor of OS in the SEER cohort

Univariate analysis in the entire cohort demonstrated age, gender, race, AJCC staging, TNM staging, primary site, tumor grade, TDs, perineural invasion, liver metastasis, lung metastasis, bone metastasis, and brain metastasis affect the patient’s OS. Moreover, multivariate analyses demonstrated that TDs were an independent prognostic factor. Using TDs-negative as a reference, patients with positive TDs represented worse OS (HR = 1.346, 95%CI: 1.296–1.398, *p* < 0.001, Table 2).

**Table 2:** Univariate and multivariate analyses of overall survival for the SEER cohort

Variable	Univariate Cox analysis		Multivariate Cox analysis	
	HR(95% CI)	*p* value	HR(95% CI)	*p* value
Age				
< 65	1 [Reference]		1 [Reference]	
≥ 65	1.936 (1.879–1.995)	< 0.001	2.362 (2.291–2.436)	< 0.001
Sex				
Male	1 [Reference]		1 [Reference]	
Female	1.052 (1.024–1.081)	< 0.001	1.104 (1.075–1.135)	< 0.001
Race				
Other	1 [Reference]		1 [Reference]	
White	1.260 (1.194–1.329)	< 0.001	1.245 (1.180–1.314)	< 0.001
Black	1.440 (1.350–1.535)	< 0.001	1.497 (1.404–1.596)	< 0.001
AJCC				
I	1 [Reference]		1 [Reference]	
II	1.642 (1.568–1.720)	< 0.001	0.962 (0.881–1.051)	0.393
III	2.333 (2.231–2.439)	< 0.001	1.277 (1.147–1.423)	< 0.001
IV	7.674 (7.325–8.041)	< 0.001	2.836 (2.532–3.176)	< 0.001
T stage				
T1	1 [Reference]		1 [Reference]	
T2	1.369 (1.278–1.467)	< 0.001	1.220 (1.138–1.309)	< 0.001
T3	2.416 (2.281–2.559)	< 0.001	1.696 (1.548–1.859)	< 0.001
T4	5.258 (4.948–5.586)	< 0.001	2.647 (2.408–2.909)	< 0.001
N stage				< 0.001
N0	1 [Reference]		1 [Reference]	
N1	1.663 (1.609–1.718)	< 0.001	0.956 (0.885–1.032)	0.248
N2	3.221 (3.117–3.328)	< 0.001	1.365 (1.266–1.472)	< 0.001
Primary site				
Rectum	1 [Reference]		1 [Reference]	
Colon	1.326 (1.280–1.373)	< 0.001	1.064 (1.026–1.103)	0.001
Grade				< 0.001
I	1 [Reference]		1 [Reference]	
II	1.289 (1.215–1.366)	< 0.001	1.023 (0.965–1.086)	0.445
III	2.225 (2.089–2.369)	< 0.001	1.284 (1.204–1.370)	< 0.001
IV	2.554 (2.353–2.773)	< 0.001	1.402 (1.289–1.524)	< 0.001
TDs				
Negative	1 [Reference]		1 [Reference]	
Positive	2.611 (2.526–2.699)	< 0.001	1.346 (1.296–1.398)	< 0.001
Perineural invasion				
Negative	1 [Reference]		1 [Reference]	
Positive	2.143 (2.068–2.220)	< 0.001	1.171 (1.127–1.216)	< 0.001
Liver metastasis				
Negative	1 [Reference]		1 [Reference]	
Positive	4.273 (4.126–4.425)	< 0.001	1.318 (1.242–1.399)	< 0.001
Lung metastasis				
Negative	1 [Reference]		1 [Reference]	
Positive	4.232 (3.955–4.529)	< 0.001	1.226 (1.139–1.319)	< 0.001
Bone metastasis				
Negative	1 [Reference]		1 [Reference]	
Positive	5.223 (4.422–6.168)	< 0.001	1.368 (1.154–1.622)	< 0.001
Brain metastasis				
Negative	1 [Reference]		1 [Reference]	
Positive	6.603 (4.974–8.767)	< 0.001	1.935 (1.452–2.579)	< 0.001

### TDs was an independent risk factor for distant metastasis in SEER and The First Affiliated Hospital of Dalian Medical University cohort

**Fig. 3:**
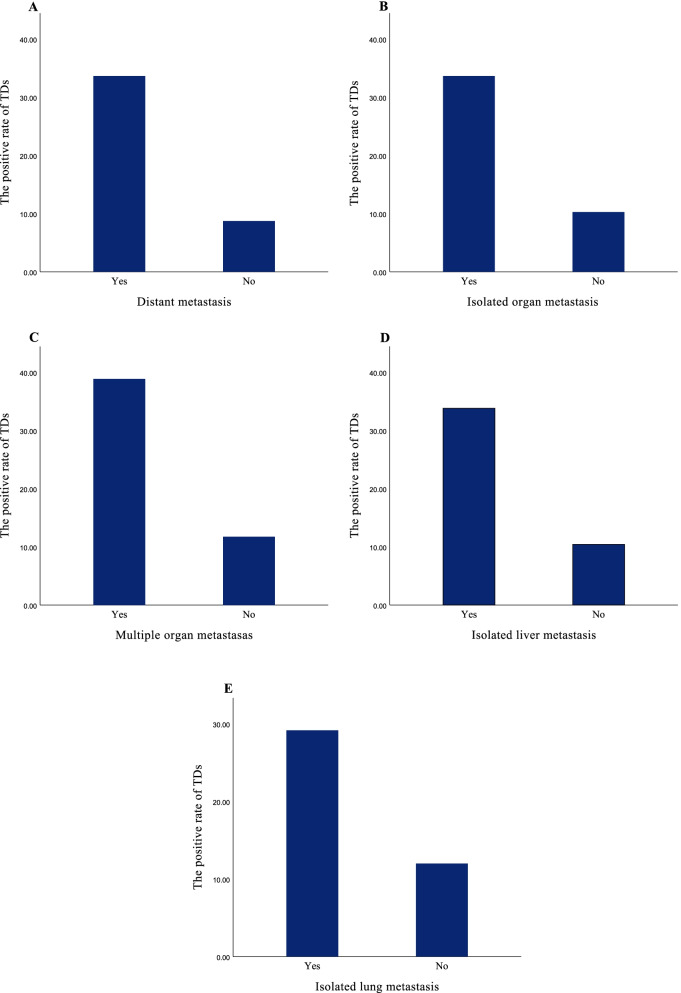
The positive rate of TDs based on absence or presence of metastasis in different patterns and verification of the distribution of TDs by chi-square test

In order to study the relationship between TDs and distant metastasis, we compared the positive rates of TDs in various metastasis patterns. The results showed that the positive rates of TDs in distant metastasis cohort (CRC tumors were observed in at least one organ), isolated organ metastasis cohort, multiple organ metastases cohort (CRC tumors were observed in at least two organs), isolated liver metastasis cohort, and isolated lung metastasis cohort were 37.28%, 33.65%, 38.85%, 33.81%, 29.16%, respectively. This was significantly higher than the TDs positive rate in the SEER cohort. Moreover, the chi-square test showed that the distribution of TDs in the above cohorts was statistically significant (*p*< 0.001, Fig. 3).

We further performed univariate and multivariate logistic regression analyses on variables in the two large cohorts to investigate the risk factors affecting patients with distant metastasis. Univariate logistic analyses revealed that TDs were an independent predictor of liver metastasis [*p* < 0.001; odds ratio (OR): 5.738; 95% confidence interval (CI): 3.560–9.248] in The First Affiliated Hospital of Dalian Medical University’s patients. Meanwhile, TDs was also an independent predictor of isolated organ metastasis [*p* < 0.001; odds ratio (OR): 3.028; 95% confidence interval (CI): 2.414–3.797]; multiple organ metastases [*p* < 0.001; odds ratio (OR): 4.778; 95% confidence interval (CI): 4.109–5.556]; isolated liver metastasis [*p* < 0.001; odds ratio (OR): 4.395; 95% confidence interval (CI): 4.099–4.713] and isolated lung metastasis [*p* < 0.001; odds ratio (OR): 5.738; 95% confidence interval (CI): 3.560–9.248] in the SEER database. Multivariate analyses suggested TDs were an independent adverse prognostic factor for distant metastasis (*p* < 0.001, Table 3).

**Table 3:** Univariate and multivariate logistic analyses of different metastatic patterns for the SEER and Dalian cohort

Distant metastasis patterns	Isolated organ metastasis OR (95% CI )	Multiple organ metastases OR (95% CI )	Isolated liver metastasis OR (95% CI )	Isolated lung metastasis OR (95% CI )	Liver metastasis OR (95% CI )
Univariate analysis TDs (+) vs TDs (−)	4.375 (4.091–4.680)	4.778 (4.109–5.556)	4.395 (4.099–4.713)	3.028 (2.414–3.797)	5.738 (3.560–9.248)
Multivariate analysis TDs (+) vs TDs (−)	1.633 (1.514–1.761)	1.667 (1.414–1.966)	1.633 (1.510–1.766)	1.402 (1.093–1.799)	4.662 (2.743–7.923)

### Some TDs-positive patients have a similar OS to stage IV patients in the SEER cohort.

**Fig. 4:**
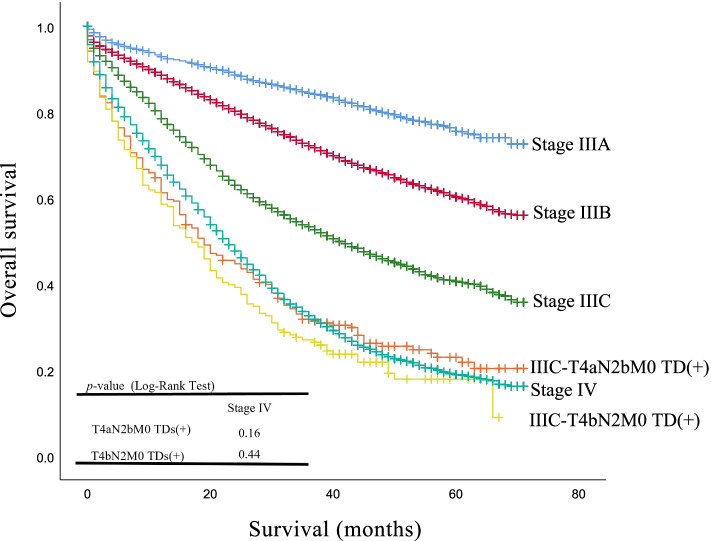
The Kaplan-Meier and log-rank test of overall survival (OS) based on the clinical stage. Note the survival curve of the “T4aN2bM0 TDs (+)” and “T4bN2M0 TDs (+)” group, which shows decreased survival compared with clinical stage III, and it is similar to the stage IV group

We wondered whether some stage III TDs positive patients were already showing similar outcomes to stage IV patients? We performed a survival analysis for each subcategory of stage III and stage IV patients; survival information is shown in Table 4 and Fig. 4, where T4aN2bM0 TDs (+) and T4bN2M0 TDs (+) patients showed the average survival period similar to patients in stage IV (28.8, 24.8, and 29.3 months, respectively) and different to those in stage IIIc (41.5 months), stage IIIb (52.7 months), and stage IIIa (60.3 months) (*p* < 0.001).

**Table 4:** Survival Analysis according to clinical stage in the SEER cohort

Clinical stage	Mean survival (months)	95% confidence interval
Stage IIIa	60.259	59.393–61.124
Stage IIIb	52.739	52.286–53.191
Stage IIIc	41.481	40.584–42.378
T4aN2bM0 TDs (+)	28.796	25.541–32.052
T4bN2M0 TDs (+)	24.789	26.132–27.261
Stage IV	29.355	28.616–30.094

## Discussion

Gabriel et al. first reported TDs in rectal cancer patients in 1935, believing that it was blood-derived metastasis confined to the surrounding tumor rather than a lymph node metastasis [18]. Goldstein et al. conducted postoperative pathology biopsies of 418 patients with T3N+M0 colorectal cancer. They found that TDs were usually distributed in large blood vessels, perinerves, or blood vessels near the primary tumor and formed when the tumor extended beyond the muscle layer. They are different from lymph node metastasis and should be described separately from lymph node metastasis [14]. This may help explain the correlation between TDs and patients' short survival time and their susceptibility to intraperitoneal metastasis. Yamano et al. divided TDs into infiltrating TDs (iTDs: cancer cell aggregates with lymphatic or perineural infiltration or cancer cell clusters) and nodular TDs (nTDs: smooth or irregularly shaped cancer cells without iTDs), found that iTDs and nTDs are independent poor prognostic factors for recurrence-free survival in patients with lymph node metastasis, and colorectal cancer patients with positive iTDs often have liver metastasis, and the probability of transition to distant lymph nodes is higher than that of patients with positive nTDs. This finding suggests that tumor cells in iTDs may transfer to the liver through the portal vein system in patients with colorectal cancer and then to lymph nodes far away from the liver [19].

This study aims to clarify the effect of TDs on the prognosis of colorectal cancer patients, including the occurrence of distant metastasis and death. We found that for overall patients, TDs-positive patients had poor OS, which was similar to the results reported in the previous study [7, 15, 16, 20]. We also found that TDs remained an independent prognostic factor for survival in patients with distant metastasis. Among stage III patients, there are still some patients with positive TDs whose prognosis are similar to that of stage IV patients. TDs can be used as an indicator of distant metastasis in this part of patients. However, the latest version of TNM staging only considers TDs without lymph node metastasis, which may lose valuable information. For patients with both lymph node metastasis and TDs positive, it is not clear whether TDs have an adverse effect on prognosis and should be included in TNM staging. In addition, there is growing support for the inclusion of TDs in category M rather than N or T in TNM staging [21–23]. The current version of the TNM staging does not mention the sites of TDs, but Yagi et al. emphasized the clinical significance of TDs in the tubercle area of the pelvis. According to the metastatic status of the LPLN (lateral pelvic lymph node) area, they divided 172 patients with stage II and III rectal cancer into three groups: patients without lymph node metastasis (no-LP-M group), patients with lymph node metastasis (LP-LNM group), and patients with TDs but without lymph node metastasis (LP-EX group). Multivariate Cox regression analysis showed that LP-EX is an important prognostic factor affecting OS and RFS, and the initial distant recurrence rate of LP-EX group (9/14, 64.3%) was significantly higher than other groups (42/158, 26.6 %) (*P* = 0.006), indicating that TDs in extrapelvic lymph node area may be a systemic disease rather than a local disease [24]. Tong et al. found that the prognosis of TDs-positive and negative colorectal cancer patients with T3N1cM0 stage was significantly different (*P* = 0.038), and it was assumed that TDs in more than 7 lymph node metastases at the same time might be similar to cases with distant metastasis. The prognosis of these cases should be attributed to stage IV [23]. Leonardo et al. conducted a cross-sectional study on 392 patients with colon adenocarcinoma and grouped patients with stage I–III with TDs as “stage IV-TD.” According to statistical analysis, the average survival time of these patients was similar to that of patients in stage IV (69.3 months vs 64.6 months) but was different from that of patients in other stages (*P* < 0.001). It can be seen that the current staging method does not fully consider the difference in the prognostic impact of TDs [25].

Although this study did not prove that TDs are directly related to stage IV patients, it is concluded that TDs are a risk factor for distant metastasis in CRC patients. Based on the above research, we should reconsider the meaning of TDs. The existence and number of TDs are related to CRC patients’ recurrence, metastasis, and survival. The greater the number of TDs, the worse the patient’s prognosis [26]. TDs have an excellent guiding significance for follow-up treatment in the long term. XiaoLi et al. found that TDs positive stage III CRC patients had a poor prognosis and did not show that DFS benefited from chemotherapy. Therefore, for TDs-positive patients, more detailed surgery and more rigorous follow-up are needed and further research on optimal treatment strategies [27]. Currently, TDs are identified by pathological slides after surgery. Due to the lack of strict pathological examination, the rate of lymph node dissection varies with the quality of the operation, and the detection of TDs has great heterogeneity, which leads to the difference in the prevalence of tumor deposition between the two cohorts. Although the latest advances in imaging have allowed MRI to detect TDs, it still takes a long time before it can be used in clinical practice [28]. The circulating tumor cells (CTC), called "liquid biopsy," have always attracted much attention from scholars. CTC refers to the heterogeneous tumor cells released from the primary tumor or metastases into the peripheral blood circulation due to spontaneous diagnosis and treatment operations, and can be detected in the peripheral blood of patients [29]. As mentioned above, TDs are closely related to distant metastases, but after surgical resection, they lose meaning in subsequent treatment and monitoring. As a more sensitive predictor, CTC has great practical significance for monitoring tumor recurrence and metastasis and treatment response [30]. Therefore, we can focus on patients with positive TDs after surgery and guide the follow-up treatment of patients by detecting the count and change trend of CTC in the blood and monitoring whether the patient has recurrence and metastasis. This kind of dynamic monitoring based on molecular characteristics can promptly identify CRC patients at risk of metastasis, reduce unnecessary costs for patients, avoid the toxic side effects of related drugs and guide patients to precise treatment is an inevitable trend in future development.

This study included the SEER database and the information of colorectal cancer patients in The First Affiliated Hospital of Dalian Medical University, but there are still limitations. The data of The First Affiliated Hospital of Dalian Medical University did not contain information on lung, bone, brain metastasis, survival status, and survival time. Information such as follow-up of whether distant metastasis occurs in patients with stage I-III, the use of TDs in very advanced clinical stages, EMVI, specific chemotherapy conditions, and the treatment of metastases may lead to deviations in research results.

## Conclusions

In conclusion, colorectal cancer patients with negative TDs have better survival benefits than patients with positive TDs. And colorectal cancer patients with positive TDs are more likely to develop distant metastasis than patients with negative TDs. Patients categorized as T4aN2bM0 TDs (+) and T4bN2M0 TDs (+) have a similar prognosis as those with stage IV, and hence these patients should be classified as stage IV. Therefore, large-scale, multi-center clinical studies should be carried out to prove the relationship between TDs and metastatic colorectal cancer, and the significance of TDs in colorectal cancer should be reconsidered.

## Supplementary Information

**Additional file 1: Table S1.** The detailed TNM staging system information in colorectal cancer.

**Additional file 2: Figure S1.** The Kaplan-Meier and log-rank test of overall survival (OS) in stage I-III patients.

**Additional file 3: Figure S2.** The Kaplan-Meier and log-rank test of overall survival (OS) in isolated bone and brain metastasis cohorts.

## Data Availability

Access to the database may be obtained from the corresponding author on reasonable request. Our data are available and publicly accessible. The original data comes from the Surveillance, Epidemiology, and End Results (SEER) database and the Department of General Surgery, The First Affiliated Hospital of Dalian Medical University.

## Abbreviations

CRC: Colorectal cancer
TDs: Tumor deposits
SEER: Surveillance, Epidemiology, and End Results
AJCC: American Joint Committee on Cancer
OR: Odds ratios
HR: Hazard ratio
CI: Confidence interval
OS: Overall survival
LNM: Lymph node metastasis
iTDs: Cancer cell aggregates with lymphatic or perineural infiltration or cancer cell clusters
nTDs: Smooth or irregularly shaped cancer cells without iTDs
LPLN: Lateral pelvic lymph node
RFS: Relapse-free survival
DFS: Disease-free survival
CTC: Circulating tumor cells
EMVI: Extramural vascular invasion

## References

[1] Siegel R, Miller K, Fuchs H, Jemal A (2021). Cancer Statistics, 2021. CA Cancer J Clin.

[2] Chen W, Zheng R, Baade P, Zhang S, Zeng H, Bray F (2016). Cancer statistics in China, 2015. CA Cancer J Clin.

[3] Ye DX, Wang SS, Huang Y, Chi P (2019). A 3-circular RNA signature as a noninvasive biomarker for diagnosis of colorectal cancer. Cancer Cell Int..

[4] McClelland D, Murray G (2015). A Comprehensive Study of Extramural Venous Invasion in Colorectal Cancer. PloS one..

[5] Tian Y, Xu T, Huang J, Zhang L, Xu S, Xiong B (2016). Tissue metabonomic phenotyping for diagnosis and prognosis of human colorectal cancer. Sci Rep.

[6] Lino-Silva L, Xinaxtle D, Salcedo-Hernández R (2020). Tumor deposits in colorectal cancer: the need for a new "pN" category. Ann Transl Med.

[7] Ueno H, Mochizuki H, Shirouzu K, Kusumi T, Yamada K, Ikegami M (2011). Actual status of distribution and prognostic impact of extramural discontinuous cancer spread in colorectal cancer. J Clin Oncol.

[8] Ueno H, Hashiguchi Y, Shimazaki H, Shinto E, Kajiwara Y, Nakanishi K (2014). Peritumoral deposits as an adverse prognostic indicator of colorectal cancer. Am J Surg.

[9] Liu F, Zhao J, Li C, Wu Y, Song W, Guo T (2019). The unique prognostic characteristics of tumor deposits in colorectal cancer patients. Ann Transl Med.

[10] Wang Y, Zhang J, Zhou M, Yang L, Wan J, Shen L (2019). Poor prognostic and staging value of tumor deposit in locally advanced rectal cancer with neoadjuvant chemoradiotherapy. Cancer Med.

[11] Song Y, Gao P, Wang Z, Liang J, Sun Z, Wang M (2012). Can the tumor deposits be counted as metastatic lymph nodes in the UICC TNM staging system for colorectal cancer?. PloS one..

[12] Li J, Yang S, Hu J, Liu H, Du F, Yin J (2016). Tumor deposits counted as positive lymph nodes in TNM staging for advanced colorectal cancer: a retrospective multicenter study. Oncotarget..

[13] Yang J, Xing S, Li J, Yang S, Hu J, Liu H (2016). Novel lymph node ratio predicts prognosis of colorectal cancer patients after radical surgery when tumor deposits are counted as positive lymph nodes: a retrospective multicenter study. Oncotarget..

[14] Goldstein N, Turner J (2000). Pericolonic tumor deposits in patients with T3N+MO colon adenocarcinomas: markers of reduced disease free survival and intra-abdominal metastases and their implications for TNM classification. Cancer..

[15] Jin M, Roth R, Rock J, Washington M, Lehman A, Frankel W (2015). The impact of tumor deposits on colonic adenocarcinoma AJCC TNM staging and outcome. Am J Surg Pathol.

[16] Nagtegaal I, Tot T, Jayne D, McShane P, Nihlberg A, Marshall H (2011). Lymph nodes, tumor deposits, and TNM: are we getting better?. J Clin Oncol.

[17] Pei JP, Zhang CD, Liang Y, Zhang C, Wu KZ, Li YZ (2020). A modified pathological n stage including status of tumor deposits in colorectal cancer with nodal metastasis. Front Oncol..

[18] Gabriel WB, Dukes C, Bussey HJR (1935). Lymphatic spread in cancer of the rectum. Bri J Surg.

[19] Yamano T, Semba S, Noda M, Yoshimura M, Kobayashi M, Hamanaka M (2015). Prognostic significance of classified extramural tumor deposits and extracapsular lymph node invasion in T3-4 colorectal cancer: a retrospective single-center study. BMC Cancer..

[20] Wong-Chong N, Motl J, Hwang G, Nassif G, Albert M, Monson J (2018). Impact of tumor deposits on oncologic outcomes in stage iii colon cancer. Dis Colon Rectum..

[21] Shimada Y, Takii Y (2010). Clinical impact of mesorectal extranodal cancer tissue in rectal cancer: detailed pathological assessment using whole-mount sections. Dis Colon Rectum..

[22] Lord AC, D'Souza N, Pucher PH, Moran BJ, Abulafi AM, Wotherspoon A (2017). Significance of extranodal tumour deposits in colorectal cancer: a systematic review and meta-analysis. Eur J Cancer..

[23] Tong LL, Gao P, Wang ZN, Song YX, Xu YY, Sun Z (2012). Is the seventh edition of the UICC/AJCC TNM staging system reasonable for patients with tumor deposits in colorectal cancer?. Ann Surg..

[24] Yagi R, Shimada Y, Kameyama H, Tajima Y, Okamura T, Sakata J (2016). Clinical significance of extramural tumor deposits in the lateral pelvic lymph node area in low rectal cancer: a retrospective study at two institutions. Ann Surg Oncol.

[25] Lino-Silva L, Anchondo-Núñez P, Chit-Huerta A, Aguilar-Romero E, Morales-Soto J, Salazar-García J (2019). Stage I-III colon cancer patients with tumor deposits behave similarly to stage IV patients. Cross-section analysis of 392 patients. J Surg Oncol.

[26] Wang S, Guan X, Ma M, Zhuang M, Ma T, Liu Z (2020). Reconsidering the prognostic significance of tumour deposit count in the TNM staging system for colorectal cancer. Sci Rep.

[27] Li X, An B, Zhao Q, Qi J, Wang W, Zhang D (2018). Impact of tumor deposits on the prognosis and chemotherapy efficacy in stage III colorectal cancer patients with different lymph node status: A retrospective cohort study in China. Int J Surg..

[28] Lord AC, Graham Martínez C, D'Souza N, Pucher PH, Brown G, Nagtegaal ID (2019). The significance of tumour deposits in rectal cancer after neoadjuvant therapy: a systematic review and meta-analysis. Eur J Cancer..

[29] Micalizzi D, Maheswaran S, Haber D (2017). A conduit to metastasis: circulating tumor cell biology. Genes Dev.

[30] Agnoletto C, Corrà F, Minotti L, Baldassari F, Crudele F, Cook W, et al. Heterogeneity in circulating tumor cells: the relevance of the stem-cell subset. Cancers. 2019;11(4):483. 10.3390/cancers11040483. 10.3390/cancers11040483 PMC6521045 30959764

