# Supplementary material for: The value of tumor deposits in evaluating colorectal cancer survival and metastasis: a population-based retrospective cohort study
Source: World J Surg Oncol. 2022 Feb 21;20:41. doi: 10.1186/s12957-022-02501-9 (PMC8862372; doi:10.1186/s12957-022-02501-9)
Supplement: Supplementary file 1 — Additional file 1: Table S1. The detailed TNM staging system information in colorectal cancer. [file 12957_2022_2501_MOESM1_ESM.docx]

Additional Table 1. The current staging criteria for colorectal cancer. (AJCC 8th)

Definition of Primary Tumor (T)

| **T Category** | **T Criteria** |
| --- | --- |
| TX | Primary tumor cannot be assessed |
| T0 | No evidence of primary tumor |
| Tis | Carcinoma in situ (intramucosal carcinoma;invasion of the lamina propria or extension into but not through the muscularis mucosae) |
| Tis(LAMN) | Low-grade appendiceal mucinous neoplasm confined by the muscularis propria. Acellular mucin or mucinous epithelium may invade into the muscularis propria.T1 and T2 are not applicable to LAMN. Acellular mucin or mucinous epithelium that extends into the subserosa or serosa should be classified as T3 or T4a, respectively. |
| T1 | Tumor invades the submucosa (through the muscularis mucosa but not into the muscularis propria) |
| T2 | Tumor invades the muscularis propria |
| T3 | Tumor invades through the muscularis propria into the subserosa or the mesoappendix |
| T4 | Tumor invades the visceral peritoneum, including the acellular mucin or mucinous epithelium involving the serosa of the appendix or mesoappendix, and/or directly invades adjacent organs or structures |
| T4a | Tumor invades through the visceral peritoneum, including the acellular mucin or mucinous epithelium involving the serosa of the appendix or serosa of the mesoappendix |
| T4b | Tumor directly invades or adheres to adjacent organs or structures |

Definition of Regional Lymph Node (N)

| **N Category** | **N Criteria** |
| --- | --- |
| NX | Regional lymph nodes cannot be assessed |
| N0 | No regional lymph node metastasis |
| N1 | One to three regional lymph nodes are positive (tumor in lymph node measuring ≥0.2 mm) or any number of tumor deposits is present, and all identifiable lymph nodes are negative |
| N1a | One regional lymph node is positive |
| N1b | Two or three regional lymph nodes are positive |
| N1c | No regional lymph nodes are positive, but there are tumor deposits in the subserosa or mesentery |
| N2 | Four or more regional lymph nodes are positive |

Definition of Distant Metastasis (M)

| **M Category** | **M Criteria** |
| --- | --- |
| M0 | No distant metastasis |
| M1 | Distant metastasis |
| M1a | Intraperitoneal acellular mucin, without identifiable tumor cells in the disseminated peritoneal mucinous deposits |
| M1b | Intraperitoneal metastasis only, including peritoneal mucinous deposits containing tumor cells |
| M1c | Metastasis to sites other than peritoneum |

AJCC PROGNOSTIC STAGE GROUPS

| **When T is…** | **And N is…** | **And M is…** | **And grade is…** | **Then the stage group is…** |
| --- | --- | --- | --- | --- |
| Tis | N0 | M0 | 0 |  |
| Tis(LAMN) | N0 | M0 | 0 |  |
| T1 | N0 | M0 | I |  |
| T2 | N0 | M0 | I |  |
| T3 | N0 | M0 | IIA |  |
| T4a | N0 | M0 | IIB |  |
| T4b | N0 | M0 | IIC |  |
| T1 | N1 | M0 | IIIA |  |
| T2 | N1 | M0 | IIIA |  |
| T3 | N1 | M0 | IIIB |  |
| T4 | N1 | M0 | IIIB |  |
| Any T | N2 | M0 | IIIC |  |
| Any T | Any N | M1a | IVA |  |
| Any T | Any N | M1b | G1 | IVA |
| Any T | Any N | M1b | G2, G3, or GX | IVB |
| Any T | Any N | M1c | Any G | IVC |
