# Supplementary material for: The value of tumor deposits in evaluating colorectal cancer survival and metastasis: a population-based retrospective cohort study
Source: World J Surg Oncol. 2022 Feb 21;20:41. doi: 10.1186/s12957-022-02501-9 (PMC8862372; doi:10.1186/s12957-022-02501-9)

Additional figure 1. The Kaplan-Meier and log-rank test of overall survival (OS) in satge I-III patients.

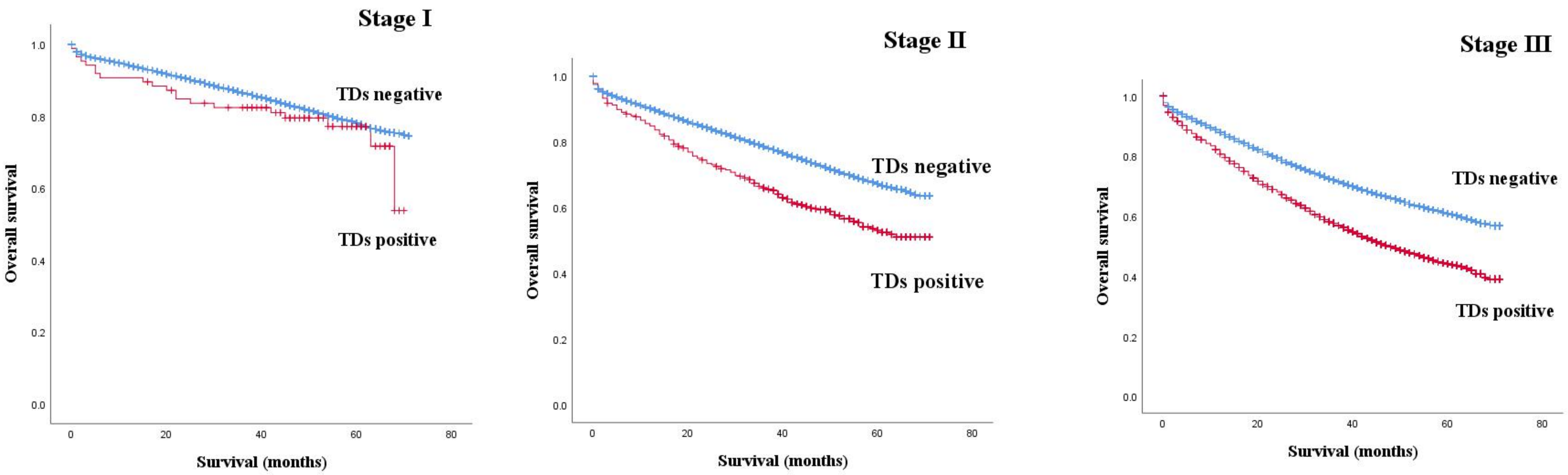

Supplement: Supplementary file 2 — Additional file 2: Figure S1. The Kaplan-Meier and log-rank test of overall survival (OS) in stage I-III patients. [file 12957_2022_2501_MOESM2_ESM.pdf]
